# Coordination of leaf functional traits under climatic warming in an arid ecosystem

**DOI:** 10.1186/s12870-022-03818-z

**Published:** 2022-09-14

**Authors:** Hongying Yu, Yingting Chen, Guangsheng Zhou, Zhenzhu Xu

**Affiliations:** 1 State Key Laboratory of Vegetation and Environmental Change, Institute of Botany, Chinese Academy of Sciences, Beijing, 100093 China; 2 State Key Laboratory of Severe Weather, Chinese Academy of Meteorological Sciences, Beijing, 100081 China; 3 University of Chinese Academy of Sciences, Beijing, 100049 China; 4 Jiyang College of Zhejiang Agriculture and Forestry University, Zhuji, 311800 China

**Keywords:** Desert steppe, Leaf economics spectrum, Key species, Climatic warming, Leaf functional traits

## Abstract

**Background:**

Climatic warming is increasing regionally and globally, and results concerning warming and its consequent drought impacts have been reported extensively. However, due to a lack of quantitative analysis of warming severities, it is still unclear how warming and warming-induced drought influence leaf functional traits, particularly how the traits coordinate with each other to cope with climatic change. To address these uncertainties, we performed a field experiment with ambient, moderate and severe warming regimes in an arid ecosystem over 4 years.

**Results:**

Severe warming significantly reduced the specific leaf area and net photosynthetic rate with a relatively stable change and even enhancement under moderate warming, especially showing species-specific performance. The current results largely indicate that a coordinated trade-off can exist between plant functional traits in plant communities in a dryland ecosystem under ambient temperature conditions, which is strongly amplified by moderate warming but diminished or even eliminated by severe warming. Based on the present findings and recent results in the relevant literature, we advance the ecological conceptual models (e.g., LES and CSR) in the response to climatic warming in arid grassland communities, where the few key species play a crucial role by balancing their functional performances to cope with environmental change.

**Conclusion:**

Our results highlight the importance of coordination and/or trade-off between leaf functional traits for understanding patterns of climatic change-induced vegetation degradation and suggest that the plant community composition in these drylands could be shifted under future climate change.

**Supplementary Information:**

The online version contains supplementary material available at 10.1186/s12870-022-03818-z.

## Background

Climatic warming is continually accelerating, profoundly impacting terrestrial ecosystems, particularly in arid areas [1–3]. The degradation of ecosystem functioning is exaggerated in drylands such as the desert steppe due to the high vulnerability to climate change [2, 4, 5]. As plant responses are heavily dependent on local meteorological variables, the field experimental simulation of climatic warming, particularly with regional heat wave features and/or various warming regimes, is a prerequisite for assessing and projecting the impacts of global environmental change on terrestrial ecosystems [6–8]. Further evaluating their cumulative effects on plants can provide a more fundamental view of ecosystem vulnerability to climate change, which can better inform policy decisions aimed at protecting natural vegetation and promoting sustainable development of terrestrial ecosystems [9–13].

High temperatures above the optimum for plant growth could negatively affect photosynthetic activity [14], carbohydrate distribution [15], nutrition stoichiometry [16], nutrient uptake, water use efficiency (WUE) and nitrogen use efficiency (NUE) [17]. Both rising temperature and drought could increase the nitrogen (N) concentration by reducing carbon accumulation in plants alone [18]; however, their combination may constrain the N level by reducing the N-absorbing capacity through root systems and N-translocating capacity in plants [14, 19, 20]. For plants in mesic and cold environments, moreover, the potential benefits of climate warming on photosynthesis can be reversed by water deficit [21], consequently exacerbating the adverse effects of high temperature [12, 14]. Nevertheless, the plant ecophysiological responses of the dominant species with different functional traits to a combination of high temperature and warming-induced drought have not been well reported, especially with regard to the underlying mechanism under field climatic warming conditions.

Leaf functional traits of the leaf economics spectrum (LES) mainly include three components: leaf structural property (e.g., SLA), leaf nutrition status (e.g., N concentration), and leaf physiological activity (e.g., light-saturated photosynthetic rate, *A*_sat_) [22]. LES reflects trade-offs between plant growth rate/leaf photosynthetic rate, construction costs, resource conservation/acquisition, and leaf longevity in plants under evolutionary and environmental drivers [23–30]. Plant species with higher SLA often have low-density leaves, higher N_mass_ and PNUE, and shorter leaf lifespans, hence contributing to faster growth rates given ample availabilities of resources [22, 31–34]. Responses to warming depend on the functional traits of different species in different biomes (e.g., [35–37]). Across global biomes, tree species with greater leaf size and higher SLA have been found to be more susceptible to drought-induced mortality than those with lower SLA [36, 38]. Many studies have also reported results regarding the relationships between plant functional traits (e.g., [22, 33, 37]). For instance, SLA is often positively correlated with *A*_sat_ and N concentrations and is strongly dependent on species, leaf types, and soil conditions [14, 22, 37]. Onoda et al. (2004) indicated that PNUE has a negative relationship with SLA [39]. However, studies concerning the relationships between plant functional traits under diverse climatic warming scenarios are relatively scant, particularly in drylands (e.g., [2, 8, 40]).

Desert steppe, an arid ecosystem, covers 23% of the total grasslands of China. More than 50% of the ecosystem is facing severe degradation characterized by decreased vegetation productivity and depleted soil nutrients [4]. Climatic warming may have a beneficial effect on C_4_ plants [41], but drought may eliminate this benefit (e.g., [14, 42]). Relative to C_3_ plants, C_4_ plants may remain at higher photosynthetic levels with higher WUE under drought and warming conditions [41, 42], suggesting that C_4_ species may have the acquisitive traits of LES [25, 41]. However, it is unknown whether and how different warming regimes and the consequent drought affect the relationships between functional traits of different species and plant functional types (PFTs) differently and how LES is involved (e.g., [42–45]). Here, we used a field infrared heating facility to simulate three climatic warming regimes in the desert steppe ecosystem to explore the effects of warming on the leaf functional traits of the key plant species, especially their relationships. We especially aimed to determine whether and how the trade-off between plant functional traits following LES in plant communities in the dryland ecosystem occurs under ambient temperature conditions, which may be strongly amplified by moderate warming but diminished or even eliminated by severe warming.

## Results

### Effects of climatic warming on leaf functional traits

Based on ANOVA, warming treatments exerted significant effects on most of the leaf functional traits except *N*_*mass*_ and *F*_v_*’/F*_m_ (Table 1; Table S1). Compared to ambient temperature (AM), only severe warming (SW) significantly reduced *SLA*, *A*_area_, *A*_mass_, and *PNUE* (*P* < 0.05). However, significant increases in *N*_area_ and *Φ*_*PSII*_ were observed under moderate warming (MW).

**Table 1:** Effects of warming on leaf functional traits (mean ± SE) at plant community level, Damao Banner, Nei Mongol, China (*n* = 3)

	Control	MW	SW
*A*_area_(μmol·m^–2^·s^–1^)	**3.84 ± 0.47 a**	**4.78 ± 0.89 a**	**1.67 ± 0.32 b**
*SLA* (cm^2^·g^–1^)	**82.49 ± 2.82 a**	**75.11 ± 4.00 ab**	**71.28 ± 4.01 bc**
*PNUE* (μmol·g^–1^ N· s^–1^)	**1.08 ± 0.12 a**	**1.14 ± 0.21 a**	**0.41 ± 0.07 b**
*N*_*area*_ (g·m^−2^)	**3.63 ± 0.11 bc**	**4.51 ± 0.20 a**	**4.05 ± 0.25 ab**
*N*_*mass*_ (mg·g^−1^)	29.18 ± 1.05 a	32.34 ± 1.77 a	27.87 ± 1.78 a
*F*_v_*'/F*_m_*'*	0.67 ± 0.01 a	0.70 ± 0.01 a	0.68 ± 0.02 a
*Φ*_*PSII*_	**0.53 ± 0.01 bc**	**0.57 ± 0.01 a**	**0.56 ± 0.02 ab**
*A*_mass_ (μmol·g^–1^·s^–1^)	**0.04 ± 0.01 a**	**0.05 ± 0.01 a**	**0.01 ± 0.00 b**

Bold font and different lowercase letters indicate significant differences among different warming treatments (*p* < 0.05). AM, MW, and SW represent ambient temperature as control, moderate warming and severe warming, respectively

Elevating temperature tended to reduce *A*_area_ and *PNUE* for the typical species except *N. pectinate* (Table 2). For *C. squarrosa*, warming did not affect *A*_area_ but significantly reduced *SLA* and *PNUE* (*P* < 0.05). There was an increase in leaf N concentration on the basis of either area (*N*_*area*_) or mass (*N*_*mass*_) (*P* < 0.05). SW increased both *Φ*_*PSII*_ and *F*_v_*’/F*_m_*’* (*P* < 0.05). For *T. terrestris*, warming markedly reduced *SLA*, *A*_area_, and *PNUE*. An increase in *N*_*area*_ was observed with no significant effects on *N*_*mass*_, *Φ*_*PSII*_, and *F*_v_*’/F*_m_*’*. For *S. tianschanica*, dramatic declines in *A*_area_ and *PNUE* occurred under SW; no significant effects on other functional traits were found from either MW or SW (*P* > 0.05). No significant effects on any functional trait were observed in *N. pectinate*. Species significantly affected all the functional traits across all warming treatments (Table S1).

**Table 2:** Effects of warming on leaf functional traits (mean ± SE) in the four key species, Damao Banner, Nei Mongol, China (*n* = 9)

Species	Treatment	*SLA* (cm^2^·g^–1^)	*N*_*mass*_ (mg·g^−1^)	*N*_*area*_ (g·m^−2^)	*A*_area_ (μmol·m^–2^·s^–1^)	*PNUE* (μmol· s^–1^ N·g^–1^)	*Φ*_*PSII*_ (dimensionless)	*F*_v_*'/F*_m_*'* (dimensionless)
*C. squarrosa*	AM	**85.78 ± 2.39 a**	**23.90 ± 0.66 b**	**2.83 ± 0.12 b**	2.10 ± 0.21 a	**0.75 ± 0.09 a**	**0.50 ± 0.02 b**	**0.56 ± 0.02 b**
	MW	**70.09 ± 5.28 b**	**28.37 ± 0.94 a**	**4.23 ± 0.33 a**	2.14 ± 0.65 a	**0.52 ± 0.15 ab**	**0.54 ± 0.02 ab**	**0.62 ± 0.02 ab**
	SW	**70.36 ± 7.70 b**	**26.83 ± 1.14 a**	**4.18 ± 0.47 a**	1.29 ± 0.40 a	**0.38 ± 0.14 b**	**0.57 ± 0.03 a**	**0.65 ± 0.04 a**
*T. terrestris*	AM	**119.05 ± 7.03 a**	45.74 ± 1.34 a	**3.94 ± 0.18 b**	**10.15 ± 1.63 ab**	**2.70 ± 0.43 a**	0.63 ± 0.02 a	0.79 ± 0.01 a
	MW	**100.18 ± 4.71 b**	46.46 ± 1.72 a	**4.70 ± 0.22 a**	**11.04 ± 1.73 a**	**2.45 ± 0.43 ab**	0.65 ± 0.02 a	0.76 ± 0.02 a
	SW	**107.58 ± 11.47ab**	51.84 ± 2.96 a	**4.85 ± 0.24 ab**	**2.12 ± 0.62 b**	**0.43 ± 0.11 b**	0.58 ± 0.08 a	0.74 ± 0.01 a
*S. tianschanica*	AM	69.38 ± 1.53 a	24.94 ± 0.73 a	3.60 ± 0.09 a	**3.13 ± 0.35 a**	**0.87 ± 0.10 a**	0.47 ± 0.02 a	0.67 ± 0.01 a
	MW	64.09 ± 2.79 a	23.63 ± 1.04 a	3.73 ± 0.13 a	**2.60 ± 0.55 ab**	**0.78 ± 0.15 ab**	0.51 ± 0.02 a	0.71 ± 0.02 a
	SW	66.93 ± 1.39 a	22.86 ± 0.81 a	3.43 ± 0.15 a	**1.51 ± 0.54 b**	**0.41 ± 0.12 b**	0.51 ± 0.03 a	0.68 ± 0.04 a
*N. pectinate*	AM	65.88 ± 3.69 a	29.28 ± 0.97 a	4.59 ± 0.25 a	2.42 ± 0.48 a	0.57 ± 0.13 a	0.58 ± 0.02 a	0.74 ± 0.02 a
	MW	54.44 ± 7.39 a	29.75 ± 2.41 a	5.73 ± 0.70 a	2.17 ± 1.12 a	0.44 ± 0.25 a	0.59 ± 0.03 a	0.72 ± 0.02 a
	SW	62.91 ± 8.61 a	29.99 ± 2.57 a	5.02 ± 0.94 a	2.86 ± 1.36 a	0.49 ± 0.22 a	0.65 ± 0.03 a	0.74 ± 0.03 a

Bold font and different lowercase letters indicate significant differences among different warming treatments (*p* < 0.05). AM, MW, and SW represent ambient temperature as control, moderate warming and severe warming, respectively

#### Divergent relationships between functional traits under different warming scenarios

**Fig. 1:**
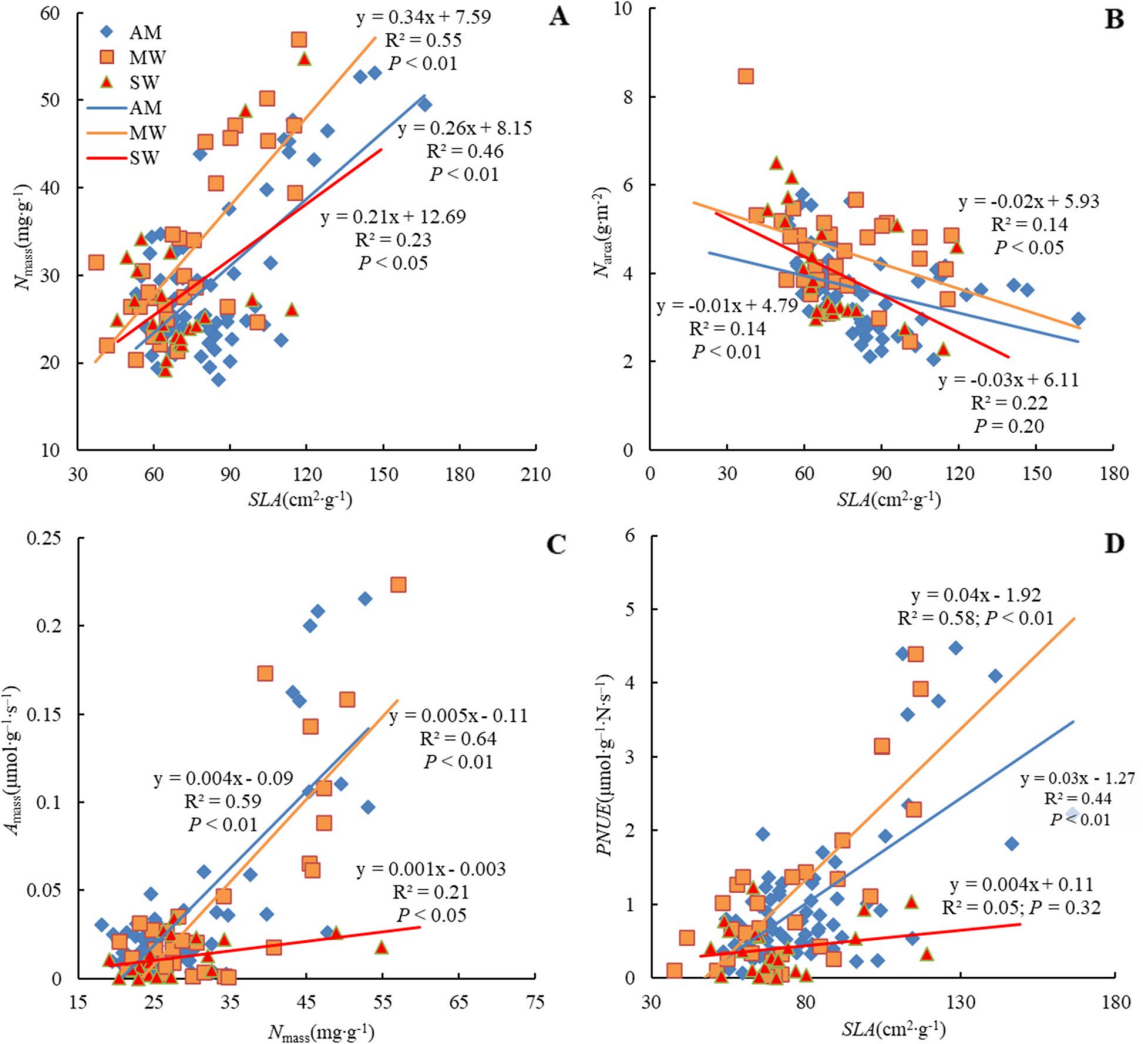
Effects of warming on relationships between *N*_mass_ and *SLA* (**A**), *N*_area_ and *SLA* (**B**), *A*_mass_ and *N*_mass_ (**C**), *PNUE* and *SLA* (**D)**. AM, MW, and SW represent ambient temperature as control, moderate warming, and severe warming, respectively

At the ecosystem level, a significant relationship occurred between *N*_*mass*_ and *SLA* across all three warming regimes (R^2^ = 0.46, *P* < 0.01 at AM; R^2^ = 0.55, *P* < 0.01 at MW; R^2^ = 0.23, *P* < 0.05 at SW; Fig. 1A). The strength of the correlation indicated that the relationship was weakened with severe warming. However, *N*_*area*_ significantly linearly decreased with *SLA* under AM and MW but not under SW (Fig. 1B). Moreover, significant and strong linear relationships between *A*_mass_ and *N*_mass_ occurred under both AM (R^2^ = 0.59, *P* < 0.01) and MW (R^2^ = 0.64, *P* < 0.01), but the relationship was weakened at SW (R^2^ = 0.21, *P* < 0.05) (Fig. 1C). Significant relationships between *PNUE* and *SLA* were also observed under both AM (R^2^ = 0.58, *P* < 0.01) and MW (R^2^ = 0.44, *P* < 0.01) but not under SW (R^2^ = 0.05, *P* = 0.32; Fig. 1D).

**Fig. 2:**
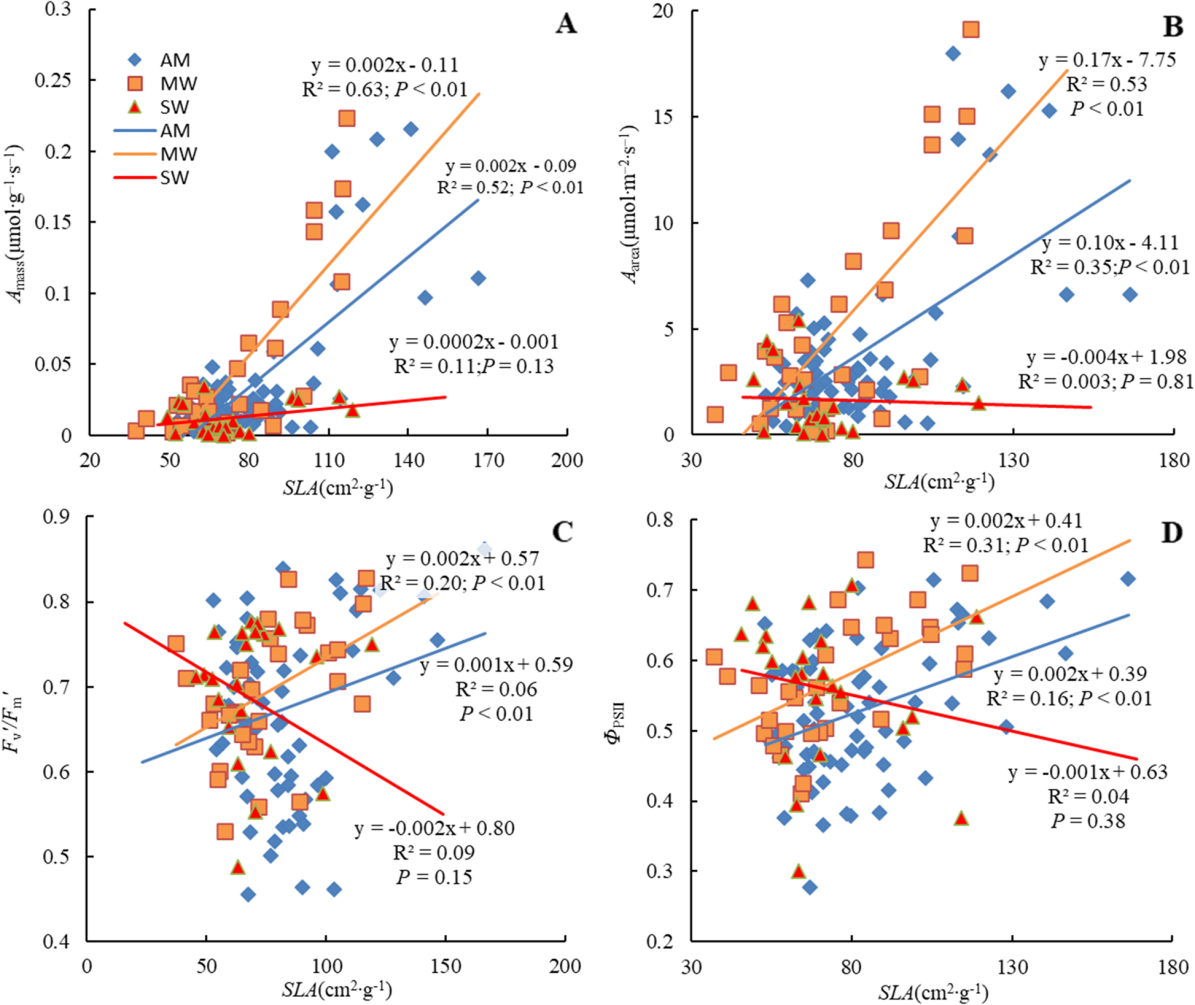
Effects of warming on relationships between *A*_mass_ and *SLA* (**A**), *A*_area_ and *SLA* (**B**), *F*_v_*'/F*_m_*'* and *SLA* (**C**), *Φ*_PSII_ and *SLA* (**D**). AM, MW, and SW represent ambient temperature as control, moderate warming and severe warming, respectively

Both *A*_mass_ and *A*_area_ significantly linearly increased with *SLA* under AM and MW but not under SW (Fig. 2AB). There were relatively weak positive relationships of both chlorophyll fluorescence parameters (i.e., *Φ*_*PSII*_ and *F*_v_*’/F*_m_*’*) with *SLA* under AM and MW but not under SW (Fig. 2CD). Under MW, the relationships between the leaf functional traits generally had higher *R*^2^ values at higher significance levels.

#### Principal component analysis of multiple functional traits

**Fig. 3:**
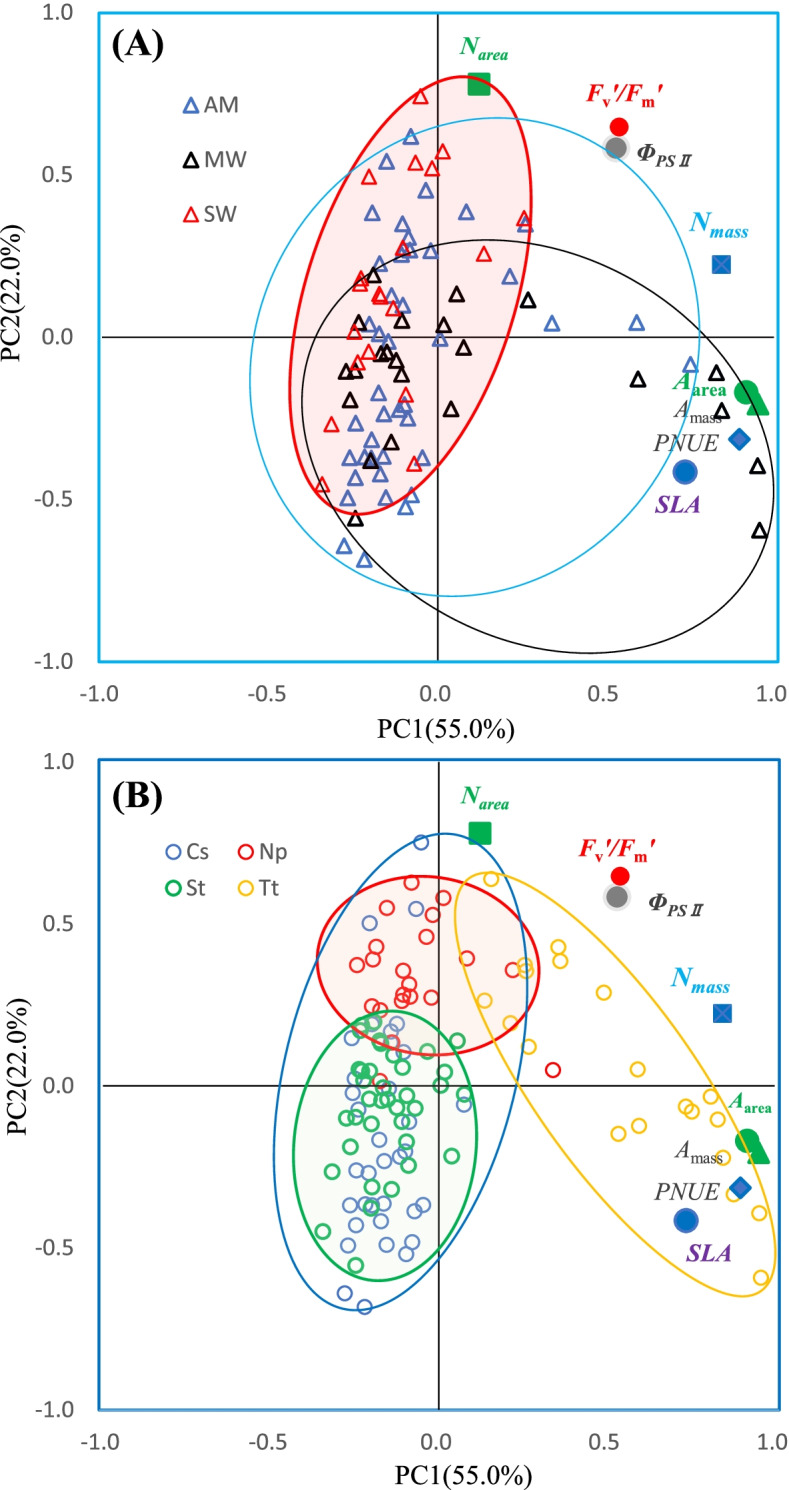
Principal component analysis on plant functional traits under the three warming regimes [ambient warming (AM), moderate warming (MW) and severe warming (SW)]. The traits’ loadings on the first two principal components (PCs) are shown, and their projections are sorted by the three warming regimes (**A**) and the four dominant species (**B)**. Loadings of leaf functional traits (see abbr. list) adjacent to solid symbols are shown. In upper panel (**A**), blue open, black open, and red open triangles represent AM, MW, and SW, respectively; while the blue, black, and red ellipses indicate the range of each treatment, accordingly. In bottom panel (**b**), blue open, green open, red open, and yellow open circles represent *C. squarrosa* (Cs), *S. tianschanica* (St), *N. pectinate* (Np), *T. terrestris* (Tt), respectively; while the blue, green, red, and yellow ellipses indicate the range of each species accordingly

The first two principal components (PC1 and PC2) represented 55.0% and 22.0%, respectively, of the total inertia, and they could jointly explain the changes in *SLA*, *N*_*mass*_, *A*_area_, and *PNUE* by 71.5, 76.4, 87.2 and 90.6%, respectively (Fig. 3A). The loadings of N concentration representing nutrient status and chlorophyll fluorescence parameters denoting PSII functioning were located in quadrant I, with the marker (SLA) representing leaf structure and net photosynthetic rate indicating gas exchange capacity in quadrant II (Fig. 3A; Table S2). The projections of the three warming regimes (Fig. 3A) and the four species (Fig. 3B) are also represented on the first two axes. Although the PC factors of AW and MW were almost scattered, the PC factors of SW were relatively contributed in the center, farther from the loadings of the leaf structure and photosynthetic rates, indicating the strong effects from SW (Fig. 3A). The PC factors of the dominant species were distinctly distributed (Fig. 3B). The PC factors of *S. tianschanica* and *N. pectinate* were relatively convergent and clustered with those of *C. squarrosa*; they were all far from the loadings of the functional traits (Fig. 3B). However, *T. terrestris* demonstrated different patterns—its PC factors seemed relatively scattered and alone but were near or across the loadings of functional traits.

#### Structural equation modeling for the causal relationships among functional traits

**Fig. 4:**
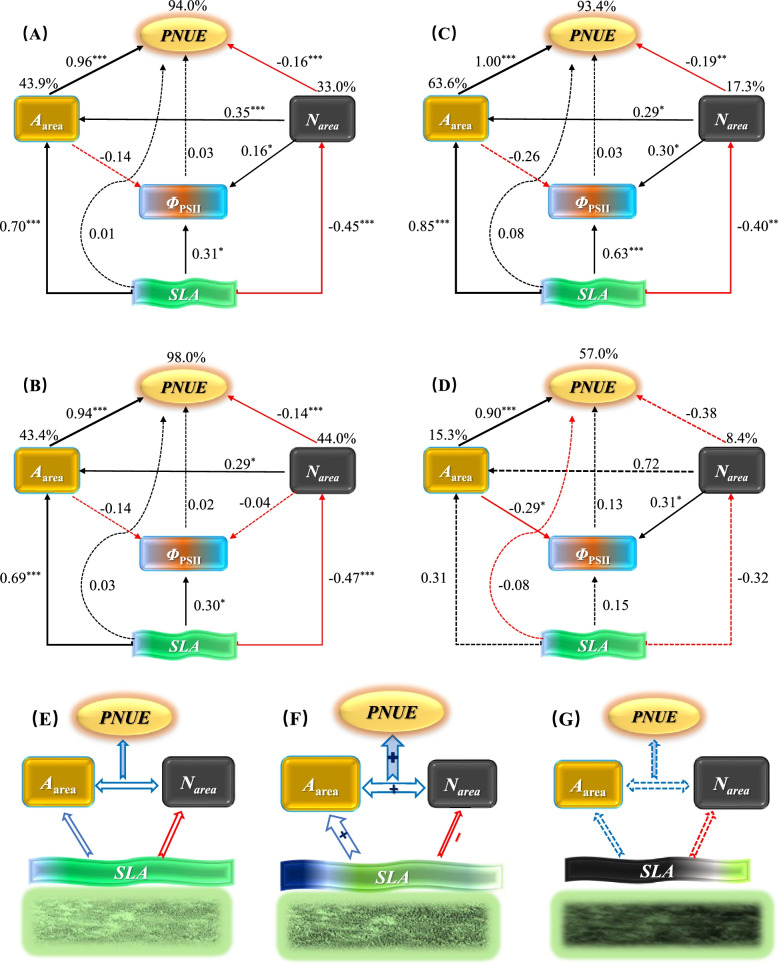
Structural equation modeling (SEM) concerning the effects of climatic warming on key leaf functional traits and their relationships across the 4-year field warming experiment (**A**, across all warming treatments; **B**, AM; **C**, MW; **D**, SW). Solid black and red arrows represent significant positive (black) or negative (red) relationships at *P* < 0.05 levels, whereas dashed black and red arrows represent no significance (*P* > 0.05). Values above arrows indicate the standard path coefficients, and their significances at 0.05, 0.01, and 0.001 levels are marked by *, **, and ***, respectively. Arrow widths are proportional to the size of the standardized path coefficient. Percentages on rectangles or ellipses indicate the variance explained by the models. Goodness-of-fit statistics are: **A** χ^2^ = 1.64; df = 3; *P* = 0.44; goodness of fit index (GFI) = 1.00; root mean square error of approximation (RMSEA) < 0.001; Akaike information criterion (AIC) = 39.64; *N* = 124. **B** χ^2^ = 0.89; df = 2; *P* = 0.65; GFI = 1.00; RMSEA < 0.001; AIC = 38.89. *N* = 68. **C** χ^2^ = 7.39; df = 2; *P* = 0.25; GFI = 0.94; RMSEA = 0.29; AIC = 45.39. *N* = 33. **D** χ^2^ = 3.62; df = 2; *P* = 0.163; GFI = 0.95; RMSEA = 0.19; AIC = 41.62. *N* = 23. Leaf economics spectrum (LES) driven by climatic warming is shown in E–F panels. Under AM, *A*_area_ increases, but *N*_area_ decreases with *SLA*, finally increasing *PNUE *(**E**). Under MW, the positive relationship of *A*_area_ with *SLA* is amplified, but that of *N*_area_ with SLA is weakened, hence enhancing *PNUE *(**F**). However, under SW, the entire relationships between the leaf functional traits were largely weakened, even decoupled, consequently terminating the LES (**G**). Blue single arrow indicates the positive response, while red single arrow indicates the negative response. Blue double arrow indicates the positive interaction, and dotted arrow represent the decreased/cancelled relationships. The + and – signs denote the amplification and reduction, respectively

Under climatic warming and soil water content (SWC) drivers separately, SLA directly positively affected *A*_area_, while it negatively affected *N*_area_. PNUE was strongly positively affected by *A*_area_ but weakly affected by *N*_area_. Warming and SWC did not significantly directly affect the functional traits alone (Fig. S1AB). However, when considering warming and SWC jointly, we found that warming had significant direct effects on SWC, *A*_area_, and *N*_area_ (*P* < 0.05). In particular, *A*_area_ and *N*_area_ were also indirectly affected by warming by affecting SWC (Fig. S1C). The SEM results demonstrated that SLA had significant positive relationships with *A*_area_, which further strongly positively affected *PNUE* across warming treatments (Fig. 4A) under AM (Fig. 4B) and MW (Fig. 4C). SLA significantly negatively affected *N*_area_, and the latter weakly negatively affected *PNUE*; SLA effects on *Φ*_PSII_ also occurred across all data and under AM and MW (Fig. 4A-C). However, under SW, these significant effects almost disappeared, and the explanations of *A*_area_, *N*_area_, and *PNUE* from the multiple traits were also diminished (Fig. 4D-G).

**Fig. 5:**
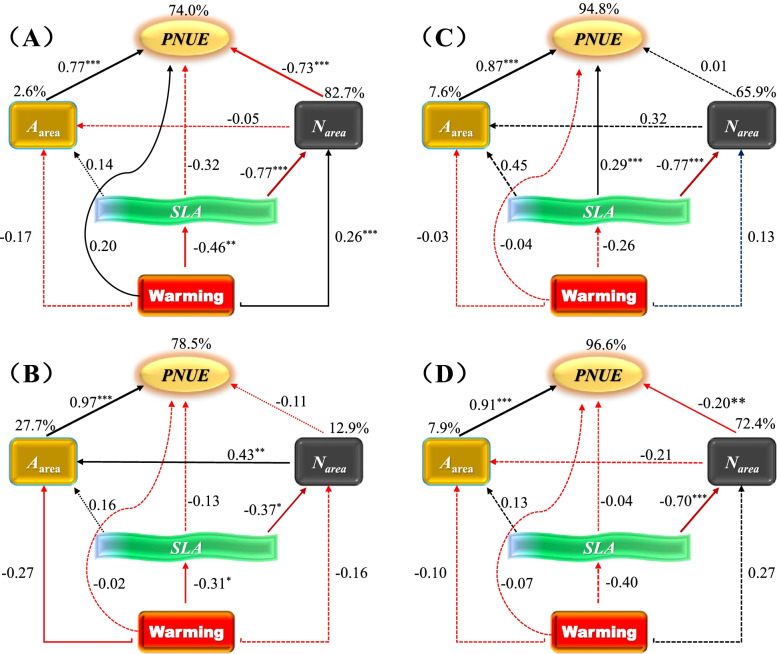
Structural equation modeling (SEM) concerning the effects of climatic warming on key leaf functional traits and their relationships across the 4-year field warming experiment for the four dominant species (**A**, *C. squarrosa*; **B**, *S. tianschanica*; **C**, *N. pectinate*; **D**, *T. terrestris*). Solid black and red arrows represent significant positive (black) or negative (red) relationships at *P* < 0.05 levels, whereas dashed black and red arrows represent no significance (*P* > 0.05). Values above arrows indicate the standard path coefficients, and their significances at 0.05, 0.01, and 0.001 levels are marked by *, **, and ***, respectively. Percentages on rectangles or ellipses indicate the variance explained by the models. Modification indices were conducted when adding paths would improve the model. The model fit is suitable: **A** χ^2^ = 2.29; df = 2; *P* = 0.27; goodness of fit index (GFI) = 0.98; root mean square error of approximation (RMSEA) = 0.009; Akaike information criterion (AIC) = 40.59; *N* = 39. **B** χ^2^ = 1.42; df = 2; *P* = 0.93; GFI = 0.99; RMSEA < 0.001; AIC = 39.42. *N* = 39. **C** χ^2^ = 0.58; df = 2; *P* = 0.75; GFI = 0.99; RMSEA < 0.001; AIC = 38.58. *N* = 23. **D** χ^2^ = 0.44; df = 2; *P* = 0.80; GFI = 0.99; RMSEA < 0.001; AIC = 38.44. *N* = 23

When considering the four dominant species separately, different patterns of SEM were observed among the species (Fig. 5A-D). For *C. squarrosa*, warming significantly negatively affected SLA, SLA similarly affected *N*_area_, and *N*_area_ similarly affected PNUE. Warming significantly negatively affected SLA, and SLA similarly affected *N*_area_, but *N*_area_ did not affect PNUE for *S. tianschanica*. Warming had no significant effects on the functional traits of either *N. pectinate* or *T. terrestris*. Significant relationships between *A*_area_ and PNUE occurred for both *C. squarrosa* and *T. terrestris*. However, a significant relationship between *A*_area_ and *N*_area_ was observed only for *S. tianschanica*, and SLA significantly affected *PNUE* only for *N. pectinate*.

## Discussion

In the field warming experiment, we found that compared with ambient temperature, severe warming significantly affected most of the leaf functional traits with highly species-specific performance. The current results indicate that a trade-off can occur between plant functional traits in plant communities in a dryland ecosystem under ambient temperature conditions, which can be enhanced by moderate warming but weakened or even canceled by severe warming. Finally, several conventional ecological models have been updated largely in response to climatic warming, where the few key species play a crucial role by mediating their functional relationships to cope with environmental change (e.g., [37, 46, 47]). The current findings suggest that using species with high resistance and resilience to high temperature and drought could address future climatic change [12, 48].

### Responses to warming and warming-induced drought

Specific leaf area (SLA) decreased under more than moderate drought stress [49, 50], while an SLA decline was observed at an elevated temperature [51]. However, SLA was also observed to increase with increasing temperature [14, 42]. It tended to decrease when plants were exposed to a combination of highly rising temperature and water deficit [42]. In the present study, a general decline with warming occurred for all four species measured (Tables 1 and 2). Moreover, the SLA declines were found to be more severe warming than moderate warming, implying that the regulatory response of SLA could be warming severity specific. In addition, high SLA variance likely reflects its functional relationship with leaf lifespan, and these perennial rather than annual plants may have large leaves with low SLA [23]. In the present study, the annual C_4_ species *T. terrestris* had the highest SLA relative to the other three perennial species, again highlighting this phenomenon.

Climatic warming could have positive [52], neutral [53], and negative [17, 54] impacts on plant nutrition status, which strongly depend on water status (e.g., [17, 53]) and ecosystem/biome types [52, 54, 55]. The present results showed no warming effect on *N*_*mass*_ across all dominant species (Table 1), indicating a neutral effect [53]. Warming may reduce the N concentration due to dilution under amply watered conditions; however, warming with drought may cancel the dilution due to inhibited plant growth [14, 56]. Thus, the current results on the N level may also complicate a trade-off strategy between warming dilution and drought denseness.

Climatic warming has also been found to have positive, neutral, and negative effects on the photosynthetic performance of plants [42, 57–59], strongly depending on species and warming regimes. In particular, both C_3_ and C_4_ species may have different photosynthetic responses to climatic warming (e.g., [57, 60]). C_4_ plants often dominate warm environments and have been suggested to have a high resistance to high temperature relative to C_3_ plants (e.g., [61, 62]). Positive responses of C_4_ species to warming have been observed in many studies (e.g., [58, 61]). However, both C_3_ and C_4_ plants have been found to have similar photosynthetic responses to warming [63, 64], and inhibition of C_4_ plants may appear with a high temperature or a combination of warming and water deficit [42, 58]. Thus, a concurrence of warming and drought jointly presenting severe abiotic stress could largely constrain the photosynthetic performance of either C_3_ or C_4_ species, especially in a xeric area [42, 58]. This was completely confirmed by the present warming experiment, where warming-induced drought stress occurred (Table 2; [5]). At higher temperatures, an increase in *A*_area_ but a decrease in *N*_*area*_ occurred in two C_4_ grasses, thus leading to a large *PNUE* increase [62]. In the current experiment, however, a slight increase in *N*_*area*_ in the two C_4_ plants was not observed (also see [14]); thus, a sharp decline in *A*_*area*_ led to a dramatic reduction in *PNUE* (Tables 1 and 2). The *PNUE* changes may also reflect the trade-off between carbon requirements and N conservation when facing climatic warming and consequently water deficits.

A high temperature above the optimum can result in a decline in *A*_area,_ as reported by a body of previous results and the present study (e.g., [14, 42, 58]; Tables 1 and 2). However, the function of PSII, using both *F*_v_*’/F*_m_*’* and *Φ*_PSII_ as the proxies [65], remained almost stable (e.g., [14, 66, 67]), and a slight promotion might even occur (Tables 1 and 2). The thermostability of PSII is strongly enhanced in drought-stressed plants [68]. Thus, compared with these gas exchange parameters, a relatively stable change or a slight decrease in *F*_v_*’/F*_m_*’* and *Φ*_PSII_ in plants exposed to climatic warming may imply that the thermostability of PSII can be improved by warming-induced drought.

### Functional trait relationships

SLA is closely related to resource availability, such as nutrients, water, heat, and light (e.g., [69–71]). The relationship between SLA and nitrogen concentration depends on soil nutrition status, environmental factors, and plant species (e.g., [14, 72, 73]). In the current study, we found strong associations of SLA with *N*_mass_ rather than *N*_area_ (Fig. 1), meaning that larger and thinner leaves had a lower N concentration at the base of the leaf area than smaller and thicker leaves. This reflects the trade-off between leaf growth and N investment [39, 73]. Lower SLA is generally associated with higher natural resource efficiency, such as WUE and NUE, under stressful conditions and thus can be recognized as a strategy of phenotypic regulation [38, 74]. A decline in SLA with water deficit and an increase with rising temperature suggests that warming may shrink the leaf structural adaptative response to drought, which may be linked to PNUE involvement [14]. Onoda et al. [39] found that PNUE is significantly and negatively related to SLA. However, the present findings indicated close relationships between functional traits under AM and MW conditions, but this relationship almost disappeared under SW, implying a shift in the relationship direction under a more stressful environment. The underlying mechanisms can be explained as follows: i) severe drought induced by SW could constrain the change in the photosynthetic rate at a low level by damaging mesophyll cell ultrastructure and enhancing lipid peroxidation, consequently weakening the relationship (e.g., less change in *A*_sat_ with SLA [75]; ii) plant growth and carbon allocation could be severely affected by severe stress, inhibiting leaf carbon investment and slowing the change in SLA [e.g., [42]]; and iii) plant N absorption, transport and allocation could also be constrained [14, 76], lessening flexible changes in leaf N concentration. Together, this may explain why severe warming diminishes the trade-off between plant functional traits. This finding implies that plant adaptative capacity will be weakened by decoupling the relationship between functional traits if climatic warming is accelerated further. Enhancing the coordination among functional traits by selecting the key species in response to climatic change may improve the sustainable management of arid ecosystems.

Compared with coastal plant species, desert species with high heat and drought tolerance and higher *F*_*v*_*/F*_*m*_ had lower SLA and smaller leaf areas [70]. However, the present results indicated that *A*_sat_ rather than *F*_*v*_*/F*_*m*_ positively correlated with SLA, again indicating the different performances between the two traits (i.e., *A*_sat_ and *F*_*v*_*/F*_*m*_) representing the photosynthetic potentials (also see [67]), highlighting the need for caution when using different functional traits in response to environmental change.

### Leaf economics spectrum (LES) under climatic warming

The current results suggest that plants can cope well with the impacts of climatic warming in terms of LES theory but only when undergoing moderate climatic stress; the positive effect of the synergistic coordination between the traits is likely to attenuate, collapse, and even reverse under severe climatic harshness [40]. In addition, the current results from PCA illustrated that AM and MW had a similar pattern affecting the leaf functional traits (Fig. 3A). Furthermore, we found that the four key dominant species—each of which can be used as a proxy for PFTs—play crucial roles in forming LES plane patterns in response to climatic warming. For instance, on PCA plane B, the dimension (upper left to lower right, ‘N_mass_–SLA’, the former is more strongly associated with PC2 than PC1, whereas the latter reverses the trend) ranges from species with conservative leaves (low-SLA, nitrogen-poor) to species with acquisitive leaves (nitrogen-rich, high-SLA, high-*A*_area_) that would have a shorter leaf lifespan and lower survival when facing environmental stress [22, 28, 46, 47, 77]. The two species, *N. pectinate* and *T. terrestris,* may be proxies of the two contrasting species, conservative and acquisitive, and which one can prosper more may strongly depend on different climate change scenarios [73, 74]. The coordination and trade-offs between traits—whether and how the LES performs—may strongly depend on plant species, PFTs, ecosystems, and environmental conditions (e.g., [36, 69, 78–80]).

### Competitors-stress-ruderals theory (CSR) under climatic warming

The distribution feature on the responses of the key species from PCA may inform the CSR, a theory related to ecological and evolutionary aspects, which projects that ruderal species will prosper under more favorable conditions, such as with richer climatic and soil nutrient resources [81–83]. As previously reported, these ruderal species with easily growing and cheaply constructed traits could rapidly respond to precipitation change and nutrient status [48], but stress-tolerant species with slow growth and expensively constructed traits may remain stable, leading to a shift in plant community composition [48, 77, 84]. In the present study, the four dominant species were grouped into four ecological strategy types—*N. pectinat*e (a native species prospering casually in some years) as the competitor, *S. tianschanica* (a unique and native species that appears every year) as the stress-tolerator, *T. terrestris* (a common species that appears casually in some years) as the ruderal species, and *C. squarrosa* (a native species that can be found every year) as an intermediate ecological strategist. This is because 1) each species has functional traits representing its specific ecological strategy type (Table 2); 2) as illustrated in the PCA planes, *N. pectinat*e is at distant points, but *T. terrestris* appears in the cheaply constructed traits (e.g., *A*_area_ and *SLA*). However, *S. tianschanica* is consistently far away, and *C. squarrosa* crossed almost all functional traits. This layout sorted by species can be compared with that by the warming treatments (Fig. 3). These findings may provide updated insight into CSR schemes for natural ecology, particularly in the global warming context.

## Conclusions

In this study, a field warming experiment was conducted with ambient, moderate and severe warming regimes in an arid ecosystem over 4 years. We found that severe warming significantly affected most functional traits with species-specific performance. There was a trade-off between plant functional traits in plant communities in the arid ecosystem under ambient temperature conditions, which can be strengthened by moderate warming but weakened or even eliminated by severe warming. Based on the present findings and recent results in the relevant literature, we advanced several conventional ecological theoretical models, e.g., LES and CSR, in arid plant communities when exposed to climatic warming, in which the few key species play a critical role by balancing their functional performances to cope with climatic change. Our results especially highlight the importance of coordination and/or trade-off between leaf functional traits within and/or among the dominant species for understanding patterns of climatic change-induced vegetation degradation and suggest that plant community composition in drylands could be shifted in the future. This could be useful for assessing and projecting vegetation change and thus improving the management practices of vulnerable ecosystems in the face of climatic warming.

## Methods and materials

### Site expressions

The experiment was located in a desert steppe, Damao Banner (County), Nei Mongol, China (41°38′38.3″N, 110°19′53.3″E, 1409 m a.s.l.). This area is characterized by a typical continental climate. From the long-term climatic record (1954–2019), the mean annual temperature (MAP) was 4.3 ℃, and the mean annual precipitation (MAT) was 259.7 mm, 73% of which precipitated during the growing season (from 1 May to 30 August). The desert steppe sampled at this site was fenced and thus ungrazed since 1980. The soil is a calcic Kastanozem (chestnut) type based on the soil classification system of FAO, with a bulk density of 1.23 g·cm^−3^. The four dominant species were selected to examine the leaf functional traits: i) *Stipa tianschanica* var. klemenzii, a C_3_ perennial grass; ii) C*leistogenes squarrosa* (Trin.) Keng, a C_4_ perennial grass; iii) *Neopallasia pectinata* (Pall.) Poljakov, a C_3_ perennial shrub; and iv) *Tribulus terrestris* L., a C_4_ annual grass [5].

### Experimental design

We conducted a field warming experiment across 4-yr plant growth seasons from early May 2010 to late August 2014. With a randomized complete block design, three treatments were designed: control (ambient temperature) (AM), moderate warming (MW), and severe warming (SW). Moderate warming treatment was imposed during 2011–2014, while SW treatment was imposed only in 2014. Warmed plots were heated 24 h/day by infrared lamps (1.0 m long, 800 W) (GHT220-800; Sanyuan Huahui Electric Light Source Co. Ltd., Beijing, China) during the growing seasons from early June to late August. The infrared lamp heights above the ground surface were 1.5 m and 1.0 m under MW and SW treatments, respectively. To minimize the effects of other environmental factors, such as shading, the control plots were also placed with “dummy” heaters similar to those in the warming plots. A total of 15 experimental plots (2 m × 2 m) were made across three experimental blocks. A 1 m buffer zone in each adjacent plot was established. Field infrared heating is recognized as an appropriate facility for heat wave simulation and has been used extensively (e.g., [6, 7, 85]). Based on the recent IPPC report [1], by the end of the twenty-first century, global surface temperature is expected to increase by 2.1 °C to 3.5 °C in the intermediate scenario and by 3.3 °C to 5.7 °C under the very high greenhouse gas emissions scenario. As expected, compared to ambient temperature (e.g., the control treatment), the soil temperatures at 0–10 cm depth under MW and SW treatments were significantly elevated by 2.6 °C and 3.2 °C, respectively. Soil moisture in the 0–20 cm soil profile was significantly reduced by 14.2% and 33.7% (v/v, *P* < 0.05) in both moderate and severe warming plots, respectively, relative to control plots (Fig. S2-3).

### Soil temperature and moisture

A thermocouple (HOBO S-TMB-M006; Onset Computer Corporation, Bourne, MA, USA) was installed at a depth of 0–10 cm, and a humidity transducer (HOBO S-SMA-M005; Onset Computer Corporation) in a soil profile of 0 to 20 cm was installed to monitor the soil temperature (°C) and soil moisture (v/v) at the center of each plot. Continuous measurement data were recorded every 2 s and averaged at half-hour intervals by a data logger (HOBO H21-002; Onset Computer Corporation).

### Leaf gas exchange and chlorophyll fluorescence

Leaf gas exchange parameters were measured using a CIRAS-2 portable photosynthesis system (PP Systems, Hertfordshire, UK) with a Chl fluorescence module (CFM) on clear sky mornings (09:00–11:00 a.m.) with less than gentle wind. The reference CO_2_ concentration in the leaf chamber was kept at 380–390 μmol·mol^−1^, with a relative air humidity of 50%–70%. We set a saturated photosynthetic photon flux density of 1500 μmol·m^−2^ s^−1^. The fully expanded leaves per plant per species in each plot were placed into the cuvette, and at least three measurements were made for each species in each plot. The light-saturated photosynthetic rate (*A*_sat_), stomatal conductance (*g*_s_), and transpiration rate (*E*) were obtained. Leaf Chl fluorescence was measured simultaneously with gas exchange. Briefly, the same leaves were light-adapted at a light intensity of 1500 μmol m^−2^ s^−1^ for at least 15 min to measure steady-state fluorescence (*F*_s_) before being given a flash (5100 μmol m^−2^ s^−1^, a pulse time of 0.3 s) to measure the maximum fluorescence (*F*_m_’). Then, leaves were exposed to far-red light for 5 s to determine the minimum light fluorescence (*F*_o_’). We calculated two Chl fluorescence parameters: the effective photochemical efficiency of photosystem II (PSII) (*F*_v_’/*F*_m_’) and the quantum yield of PSII photochemistry (Φ_PSII_). These calculations were performed using the following equations [86]:

1 $${F}_{\mathtt{v}}\mathrm{^{\prime}}/{F}_{\mathrm{m}}\mathrm{^{\prime}}=({F}_{\mathrm{m}}\mathrm{^{\prime}} -{F}_{\mathrm{o}}\mathrm{^{\prime}})/{F}_{\mathrm{m}}\mathrm{^{\prime}}$$

2 $${\Phi }_{\mathrm{PSII}}=({F}_{\mathrm{m}}\mathrm{^{\prime}} -{F}_{\mathrm{s}})/{F}_{\mathrm{m}}\mathrm{^{\prime}}$$

### Leaf carbon and nitrogen

The dried leaf samples of each kay species for gas exchange measurement in each plot were mixed and ground for 1 min by a mixer mill (Retsch MM400, Germany). The carbon and N concentrations of leaf dried samples were determined by an element analyzer (Elementar Vario EL III, Germany).

### Specific leaf area

All sampled leaves were scanned for leaf area with a WinFOLIA leaf/root measurement system (Régent Instruments Inc., Canada) and weighed for leaf biomass after drying at 65 °C in an oven until a consistent weight was reached. The SLA of each species was calculated as each leaf area divided by its dry mass. The photosynthetic nitrogen use efficiency (*PNUE*; μmol CO_2_ s^−1^ N g^−1^) was calculated as *A*_sat_ divided by the N concentration on a leaf area basis [87].

### Statistical analyses

To determine the effects of climatic warming on the plant functional traits of the four dominant species, we used a mixed model ANOVA with temperature treatment as a fixed factor and block as a random factor. Duncan’s post-hoc tests were performed to test significant differences between warming treatments for all leaf functional traits of each species and across the four species and between species across warming treatments during plant growth peaking in the final year. Regressions were performed to test relationships between leaf nutrient (i.e., leaf N concentration) and photosynthetic traits (i.e., net photosynthetic rate and chlorophyll fluorescence parameters, ChlF) and the leaf structural trait (i.e., SLA) under each warming treatment across all four species. The comprehensive relationships between leaf functional traits and their responses to climatic warming scenarios and plant species were determined by principal component analysis (PCA). The statistical analyses above were conducted using the statistical software package SPSS 20.0 (SPSS Inc., Chicago, IL, USA). In addition, structural equation modeling (SEM) was used to test the direct and indirect effects of warming and it-induced drought on the key leaf functional traits and their relationships. Maximum likelihood estimation was used to fit the model. Adequate model fits were indicated by a nonsignificant chi-squared test (*p* > 0.05), goodness-of-fit index (GFI > 0.90); root mean square error of approximation (RMSEA < 0.05), and Akaike information criterion (AIC, less is better) (e.g., [46]). AMOS 21.0 statistical software was used to perform the SEM analysis (IBM Corp., Armonk, NY).

## Supplementary Information

**Additional file 1:Table S1. **A two-way ANOVA test between warming treatments and plant species.** Table S2. **Correlations between the functional traits and principal component score (PC) 1 and PC 2.** Figure S1. **Structural equation modeling (SEM) concerning the effects of climatic warming on key leaf functional traits and their relationships across the 4-year field warming experiment across all warming treatments**. Figure S2. **Warming-induced changes in daily mean soil moisture (0-20 cm, A) and daily mean soil temperature (0-10 cm, B) during the growing season in 2014.** Figure S3.** Effects of warming on soil temperature (0-10 cm) and soil moisture (0-20 cm) (mean ± SE) during growing season in 2014.

## Data Availability

The data sets supporting the results of this article are included within the article and its additional files.

## Abbreviations

*SLA*: Specific leaf area
*A*_area_: Light-saturated photosynthetic rate per unit area
*A*_mass_: Light-saturated photosynthetic rate per unit mass
*N*_area_: Leaf nitrogen concentration on area basis
*N*_mass_: Leaf nitrogen concentration on mass basis
*PNUE*: Photosynthetic nitrogen use efficiency
*F*_v_*’/F*_m_*’*: Photochemical efficiency of photosystem II (PSII) in the light
*Φ*_PS__II_: Quantum yield of PSII electron transport
