# Supplementary material for: Coordination of leaf functional traits under climatic warming in an arid ecosystem
Source: BMC Plant Biol. 2022 Sep 14;22:439. doi: 10.1186/s12870-022-03818-z (PMC9472406; doi:10.1186/s12870-022-03818-z)
Supplement: Supplementary file 1 — Additional file 1:Table S1. A two-way ANOVA test between warming treatments and plant species. Table S2. Correlations between the functional traits and principal component score (PC) 1 and PC 2. Figure S1. Structural equation modeling (SEM) concerning the effects of climatic warming on key leaf functional traits and their relationships across the 4-year field warming experiment across all warming treatments. Figure S2. Warming-induced changes in daily mean soil moisture (0-20 cm, A) and daily mean soil temperature (0-10 cm, B) during the growing season in 2014. Figure S3. Effects of warming on soil temperature (0-10 cm) and soil moisture (0-20 cm) (mean ± SE) during growing season in 2014. [file 12870_2022_3818_MOESM1_ESM.docx]

**Supplementary information**

**Coordination of leaf functional traits under climatic warming in an arid ecosystem**

by Yu et al.

**The data of the leaf functional traits in "Data set.xlsx"**

Dominant species include *Cleistogenes squarrosa* (Cs), *Stipa tianschanica* (St)*,Tribulus terrestris* (Tt), and *Neopallasia pectinate* (Np). For the treatments, 1, 2, 3 indicate ambient temperature (CK), moderate warming (MW), severe warming (SW), respectively; Life history (lifeH): 1 and 2 indicate perennial (PS) and annual (AS) species, respectively; Life form (lifeF): 1 and 2 indicate grass and shrub plants, respectively; Photosynthetic pathway (PhW): 1 and 2 indicate C_3_ and C_4_ species, respectively. LifeH, life history; PS, perennial species, AS, annual species; lifeF, life form; PhW, Photosynthetic pathway; *SLA*: specific leaf area; *A*_area_, light-saturated photosynthetic rate per unit area; *A*_mass_: light-saturated photosynthetic rate per unit mass; *N*_area_: leaf nitrogen concentration on area basis; *N*_mass_: leaf nitrogen concentration on mass basis; *PNUE*: photosynthetic nitrogen use efficiency; *F*_v_*'/F*_m_*'*: photochemical efficiency of photosystem II (PSII) in the light; *Φ*_PSⅡ_: quantum yield of PSII electron transport.

**Table S1.** A two-way ANOVA test between warming treatments and plant species.

**Table S2**.Correlations between the functional traits and principal component score (PC) 1 and PC 2.

**Figure S1.** Structural equation modeling (SEM) concerning the effects of climatic warming on key leaf functional traits and their relationships across the 4-year field warming experiment across all warming treatments

**Figure S2.** Warming-induced changes in daily mean soil moisture (0-20 cm, A) and daily mean soil temperature (0-10 cm, B) during the growing season in 2014.

**Figure S3.** Effects of warming on soil temperature (0-10 cm) and soil moisture (0-20 cm) (mean ± SE) during growing season in 2014.

**Table S1**. A two-way ANOVA test between warming treatments and plant species.

| Source | Variables | Sum of Squares | df | Mean Square | F | *P* |
| --- | --- | --- | --- | --- | --- | --- |
| Treatment | ***A*_area_** | **79.569** | **2** | **39.785** | **5.722** | **0.004** |
|  | *Φ*_PSII_ | 0.019 | 2 | 0.010 | 1.567 | 0.214 |
|  | *F*_v_*'/F*_m_*'* | 0.000 | 2 | 0.000 | .042 | 0.959 |
|  | ***SLA*** | **3671.593** | **2** | **1835.797** | **9.957** | **<0.001** |
|  | *N*_mass_ | 60.556 | 2 | 30.278 | 2.225 | 0.113 |
|  | ***N*_area_** | **16.924** | **2** | **8.462** | **13.883** | **<0.001** |
|  | ***A*_mass_** | **0.011** | **2** | **0.006** | **6.319** | **0.003** |
|  | ***PNUE*** | **7.287** | **2** | **3.644** | **7.711** | **0.001** |
| Species | ***A*_area_** | **336.866** | **3** | **112.289** | **16.150** | **<0.001** |
|  | ***Φ*_PSII_** | **0.209** | **3** | **0.070** | **11.289** | **<0.001** |
|  | ***F*_v_*'/F*_m_*'*** | **0.305** | **3** | **0.102** | **21.008** | **<0.001** |
|  | ***SLA*** | **20785.312** | **3** | **6928.437** | **37.577** | **<0.001** |
|  | ***N*_mass_** | **5598.926** | **3** | **1866.309** | **137.169** | **<0.001** |
|  | ***N*_area_** | **27.879** | **3** | **9.293** | **15.247** | **<0.001** |
|  | ***A*_mass_** | **0.057** | **3** | **0.019** | **21.689** | **<0.001** |
|  | ***PNUE*** | **17.774** | **3** | **5.925** | **12.538** | **<0.001** |
| Treatment * Species | ***A*_area_** | **104.539** | **6** | **17.423** | **2.506** | **0.026** |
|  | *Φ*_PSII_ | 0.018 | 6 | 0.003 | .496 | 0.810 |
|  | *F*_v_*'/F*_m_*'* | 0.034 | 6 | 0.006 | 1.175 | 0.325 |
|  | *SLA* | 955.290 | 6 | 159.215 | .864 | 0.524 |
|  | ***N*_mass_** | **204.269** | **6** | **34.045** | **2.502** | **0.027** |
|  | ***N*_area_** | **8.925** | **6** | **1.487** | **2.440** | **0.030** |
|  | ***A*_mass_** | **0.014** | **6** | **0.002** | **2.741** | **0.016** |
|  | *PNUE* | 6.193 | 6 | 1.032 | 2.184 | 0.050 |
| Error | *A*_area_ | 730.067 | 105 | 6.953 |  |  |
|  | *Φ*_PSII_ | 0.648 | 105 | 0.006 |  |  |
|  | *F*_v_*'/F*_m_*'* | 0.508 | 105 | 0.005 |  |  |
|  | *SLA* | 19359.621 | 105 | 184.377 |  |  |
|  | *N*_mass_ | 1428.616 | 105 | 13.606 |  |  |
|  | *N*_area_ | 64.000 | 105 | 0.610 |  |  |
|  | *A*_mass_ | 0.092 | 105 | 0.001 |  |  |
|  | *PNUE* | 49.618 | 105 | 0.473 |  |  |

Bold font parts represent significant at the 0.05 level.

**Table S2**.Correlations between the functional traits and principal component score (PC) 1 and PC 2.

|  | | *SLA* | *N*_mass_ | *N*_area_ | *A*_area_ | *A_mas_*_s_ | *PNUE* | *Φ*_PSⅡ_ | *F*_v_*'/F*_m_*'* |
| --- | --- | --- | --- | --- | --- | --- | --- | --- | --- |
| PC1 | Correlation | **0.736^**^** | **0.845^**^** | 0.123 | 0.918^**^ | 0.953^**^ | 0.898^**^ | 0.531^**^ | 0.540^**^ |
|  | *P* | **<0.001** | **<0.001** | 0.187 | <0.001 | <0.001 | <0.001 | <0.001 | <0.001 |
| PC2 | Correlation | **-0.416^**^** | **0.223^*^** | **0.779^**^** | -0.170 | **-0.207^*^** | **-0.315^**^** | **0.582^**^** | **0.647^**^** |
|  | *P* | **<0.001** | **0.016** | **<0.001** | 0.067 | **0.025** | **0.001** | **<0.001** | **<0.001** |

* and ** indicate significant at the 0.05 and 0.001 levels, respectively (N = 117). Bold font parts represent significant at the 0.05 level.

**Figure S1**. Structural equation modeling (SEM) concerning the effects of climatic warming on key leaf functional traits and their relationships across the 4-year field warming experiment across all warming treatments (A, warming effects; B, effects of soil water content (SWC); C, effects of warming and it-induced SWC). Solid black and red arrows represent significant positive (black) or negative (red) relationships at *P* < 0.05 levels, whereas dashed black and red arrows represent no significance (*P* > 0.05). Values above arrows indicate the standard path coefficients, and their significances at 0.05, 0.01, and 0.001 levels are marked by *, **, and ***, respectively. Percentages on rectangles or ellipses indicate the variance explained by the models. Modification indices were conducted when adding paths would improve the model. The model fit is suitable: (A) χ^2^ = 6.41; df = 3; probability level = 0.09; goodness of fit index (GFI) = 0.98; root mean square error of approximation (RMSEA) = 0.09; Akaike information criterion (AIC) = 42.26; *N* = 124. (B) χ^2^ = 1.33; df = 2; probability level = 0.52; GFI = 1.00; RMSEA < 0.001; AIC = 39.33. *N*= 124. (C) χ^2^ = 1.52; df = 3; probability level = 0.68; GFI = 1.00; RMSEA < 0.001; AIC = 51.52. *N*= 124.


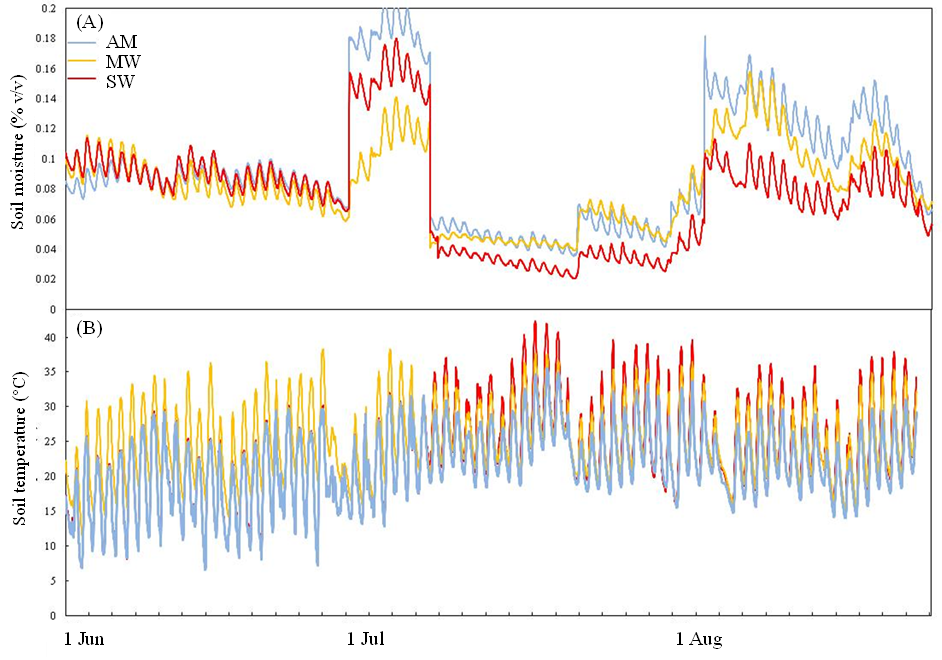


**Figure S2.** Warming-induced changes in daily mean soil moisture (0-20 cm, A) and daily mean soil temperature (0-10 cm, B) during the growing season in 2014.


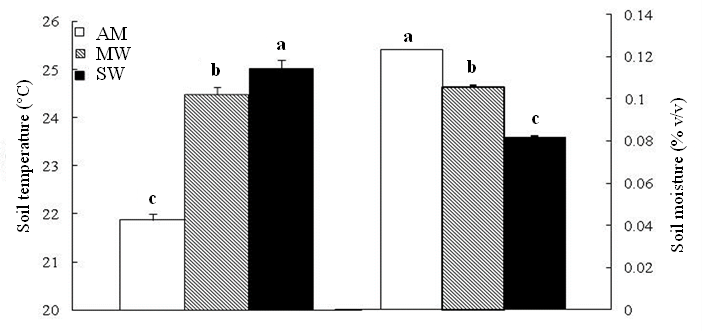


**Figure S3.** Effects of warming on soil temperature (0-10 cm) and soil moisture (0-20 cm) (mean ± SE) during growing season in 2014. Different lowercase letters indicate significant differences among different warming treatments (*p* < 0.05).
